# Regulation and Release of Vasoactive Endoglin by Brain Endothelium in Response to Hypoxia/Reoxygenation in Stroke

**DOI:** 10.3390/ijms23137085

**Published:** 2022-06-25

**Authors:** Axel Haarmann, Lena Zimmermann, Michael Bieber, Christine Silwedel, Guido Stoll, Michael K. Schuhmann

**Affiliations:** 1 Department of Neurology, University of Würzburg, 97080 Würzburg, Germany; papp_l@ukw.de (L.Z.); bieber_m@ukw.de (M.B.); stoll_g@ukw.de (G.S.); 2 University Children’s Hospital, University of Würzburg, 97080 Würzburg, Germany; silwedel_c@ukw.de

**Keywords:** endoglin, soluble endoglin, CD105, human brain endothelium, HBMEC, hypoxia, reoxygenation, ischemia/reperfusion injury, vascular homeostasis, middle cerebral artery occlusion, stroke

## Abstract

In large vessel occlusion stroke, recanalization to restore cerebral perfusion is essential but not necessarily sufficient for a favorable outcome. Paradoxically, in some patients, reperfusion carries the risk of increased tissue damage and cerebral hemorrhage. Experimental and clinical data suggest that endothelial cells, representing the interface for detrimental platelet and leukocyte responses, likely play a crucial role in the phenomenon referred to as ischemia/reperfusion (I/R)-injury, but the mechanisms are unknown. We aimed to determine the role of endoglin in cerebral I/R-injury; endoglin is a membrane-bound protein abundantly expressed by endothelial cells that has previously been shown to be involved in the maintenance of vascular homeostasis. We investigated the expression of membranous endoglin (using Western blotting and RT-PCR) and the generation of soluble endoglin (using an enzyme-linked immunosorbent assay of cell culture supernatants) after hypoxia and subsequent reoxygenation in human non-immortalized brain endothelial cells. To validate these in vitro data, we additionally examined endoglin expression in an intraluminal monofilament model of permanent and transient middle cerebral artery occlusion in mice. Subsequently, the effects of recombinant human soluble endoglin were assessed by label-free impedance-based measurement of endothelial monolayer integrity (using the xCELLigence DP system) and immunocytochemistry. Endoglin expression is highly inducible by hypoxia in human brain endothelial monolayers in vitro, and subsequent reoxygenation induced its shedding. These findings were corroborated in mice during MCAO; an upregulation of endoglin was displayed in the infarcted hemispheres under occlusion, whereas endoglin expression was significantly diminished after transient MCAO, which is indicative of shedding. Of note is the finding that soluble endoglin induced an inflammatory phenotype in endothelial monolayers. The treatment of HBMEC with endoglin resulted in a decrease in transendothelial resistance and the downregulation of VE-cadherin. Our data establish a novel mechanism in which hypoxia triggers the initial endothelial upregulation of endoglin and subsequent reoxygenation triggers its release as a vasoactive mediator that, when rinsed into adjacent vascular beds after recanalization, can contribute to cerebral reperfusion injury.

## 1. Introduction

Ischemic stroke is a major cause of death and disability. The recanalization of a cerebral large vessel occlusion (LVO) by thrombolysis and/or mechanical thrombectomy (MT) is a mainstay of acute stroke treatment, but it is still of limited therapeutic efficacy in about 50% of patients [1]. Infarct progression before and after recanalization often goes along with disturbance of the blood–brain barrier (BBB) [2,3], which increases the risk of secondary hemorrhagic transformation and unfavorable outcomes [4]. The stabilization of the BBB could therefore evolve as an adjunct treatment option. Endoglin (ENG) is a homodimeric membrane protein that is abundantly expressed by endothelial cells, including human brain endothelium [5,6,7,8]. It is closely intertwined with the maintenance of vascular integrity: the loss of ENG is embryonic lethal, and heterozygous mutations within the ENG gene cause hereditary hemorrhagic telangiectasia, a vascular disease characterized by arteriovenous malformations and bleeding complications [9,10]. Endothelial ENG is inducible by cellular stress, such as the mechanical disruption of endothelial monolayers and inflammatory cytokines [11,12,13]. In addition, ENG is upregulated in response to hypoxia in various vascular beds in vitro and, thereby, potentially protects endothelia from TGFβ-induced apoptosis [7,14]. Moreover, the membrane-bound form of ENG can be shed to a soluble isoform (sENG) by matrix metalloproteinase-12 and -14 (MMP12 and MMP14, respectively) that exerts systemic functions [15,16,17], pointing towards a possible role of sENG as a biomarker and even a potential therapeutic target for ischemia/reperfusion (I/R)-injury. However, the regulation and function of ENG in the setting of (hyper-) acute stroke during vascular occlusion and within the first hours after recanalization have been largely unexplored. Here, for the first time, we describe the upregulation of ENG in cerebral endothelia under hypoxia and the profound effect of reoxygenation that led to the shedding and release of sENG.

## 2. Results

### 2.1. Hypoxia Induces Endoglin Expression in Brain Endothelium

**Figure 1:**
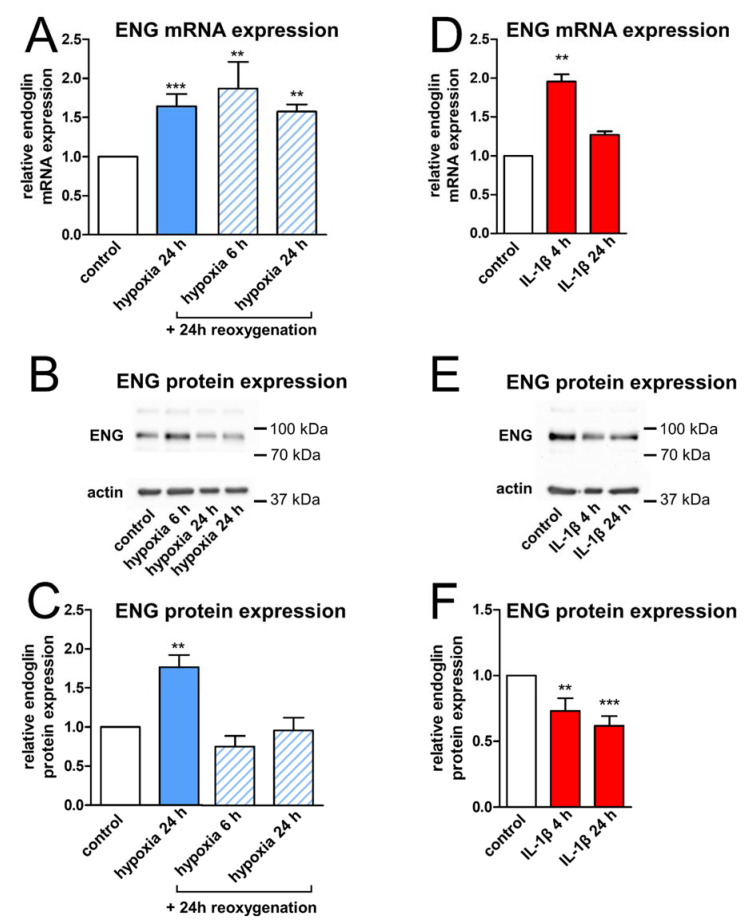
Hypoxia- and inflammation-modulated endoglin (ENG) expression in human brain endothelial cells (HBMEC). (**A**) ENG mRNA levels by quantitative real-time PCR in HBMEC after hypoxia (1% O_2_) or hypoxia/reoxygenation for the indicated durations relative to an unstimulated control. Data of six independent experiments and statistical analysis by the Kruskal–Wallis test followed by Dunn’s post-hoc test. (**B**) Representative Western blot on whole-cell extracts after stimulation as in A. (**C**) Quantification of ENG protein expression relative to β-actin in stimulated compared to unstimulated HBMEC of four independent experiments. Statistical testing for normal distribution by the D’Agostino–Pearson omnibus K2 test, followed by one-way ANOVA and the Bonferroni post-hoc test. (**D**–**F**) ENG mRNA levels and ENG protein concentrations after stimulation with IL-1β (10 ng/mL) for 4 h and 24 h. Statistics as in B. Asterisks indicate significance compared to an unstimulated control (** *p* < 0.01; *** *p* < 0.001).

First, we subjected confluent HBMEC monolayers to quantitative real-time PCR and Western blot analysis, since the upregulation of ENG after hypoxia was described in other vascular beds and post-mortem sections of stroke patients [7,14,18,19]. On the mRNA level, ENG expression was highly inducible by hypoxia (Figure 1A). Correspondingly, we found a significant increase in ENG protein levels in cells exposed to hypoxia in Western blot experiments on whole-cell lysates (Figure 1B,C). Next, in light of mechanical thrombectomy, we particularly wanted to examine whether subsequent reoxygenation additionally modifies ENG expression. While increased ENG mRNA expression persisted when hypoxia was followed by reoxygenation (Figure 1A), the ENG protein concentration after hypoxia/reoxygenation was comparable to the baseline control level, which is potentially indicative of a loss of membrane-bound ENG (Figure 1B,C). Of note stimulation of our cell model with IL-1β, shown to be upregulated in experimental and human stroke [20] and previously described as effectively inducing the shedding processes in various endothelial cells [21,22], revealed a significant decrease of −43% [0.57 (0.39–0.75)] in membrane-bound ENG protein levels after 24 h (Figure 1E,F).

### 2.2. Brain Endothelium Sheds Soluble ENG in Response to Reoxygenation

**Figure 2:**
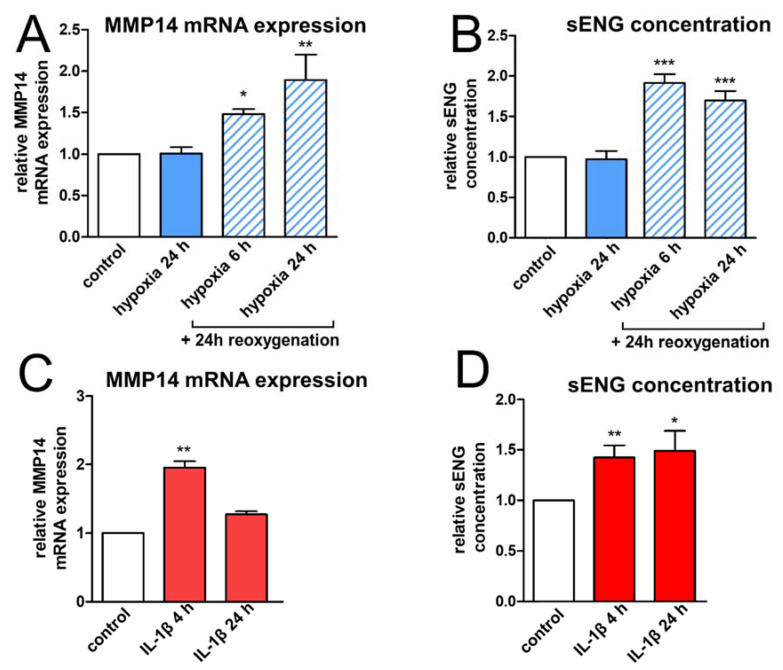
Increased matrix metalloproteinase-14 (MMP14) expression of human brain endothelium goes hand in hand with shedding of soluble endoglin (ENG). (**A**) MMP14 mRNA concentrations by quantitative real-time PCR in HBMEC after hypoxia (1% O_2_) or hypoxia/reoxygenation for the indicated durations relative to an unstimulated control. Data of three independent experiments. Statistical analysis by the Kruskal–Wallis test followed by Dunn’s post-hoc test. (**B**) Enzyme-linked immunosorbent assay for sENG in supernatants of HBMEC, stimulated as in A. Concentration relative to an untreated control. Statistical testing for normal distribution by the D’Agostino-Pearson omnibus K2 test, followed by one-way ANOVA and the Bonferroni post-test. (**C**) MMP14 mRNA concentrations after stimulation with IL-1β (10 ng/mL) for 4 h and 24 h; statistics as in A. (**D**) Concentration of sENG in cell culture supernatants after stimulation with IL-1β (10 ng/mL) for 4 h and 24 h; statistics as in B. (* *p* < 0.05; ** *p* < 0.01; *** *p* < 0.001).

Next, to unravel whether differences in ENG mRNA and protein concentrations might indeed result from shedding, we investigated the expression of MMP14, which is a membrane-bound kinase capable of cleaving the extracellular part of ENG close to the cell membrane and thereby generating a soluble isoform [23]. The sequence of hypoxia/reoxygenation persistently induced MMP14 mRNA expression; by contrast, hypoxia alone did not (Figure 2A). Analogously, IL-1β stimulation transiently upregulated MMP14 mRNA after 4 h (Figure 2C). To cross-check that the “missing” ENG was indeed present in the cell culture supernatant (i.e., it was shed), we subsequently performed enzyme-linked immunosorbent assays with cell culture supernatants of HBMEC for soluble ENG. In accordance with the increased MMP14 expression, we found a substantial release of sENG after advanced hypoxia/reoxygenation but not after hypoxia alone (Figure 2B). This shedding might be the reason that, despite ENG induction on the mRNA level, ENG protein concentrations remained stable after 6 h and 24 h of hypoxia followed by reoxygenation. With IL-1β stimulation, sENG concentration peaked after 24 h (Figure 2D).

Previous data on the release of sENG by endothelial cells are mainly based on experiments with human umbilical vein endothelial cells (HUVEC). Of note the absolute sENG concentrations detected in supernatants of HBMEC were significantly higher (5.5 ng/mL [4.6–6.4]) than in experiments with HUVEC (0.15 ng/mL [0.09–0.4]) carried out in parallel (Supplementary Figure S1).

**Figure 3:**
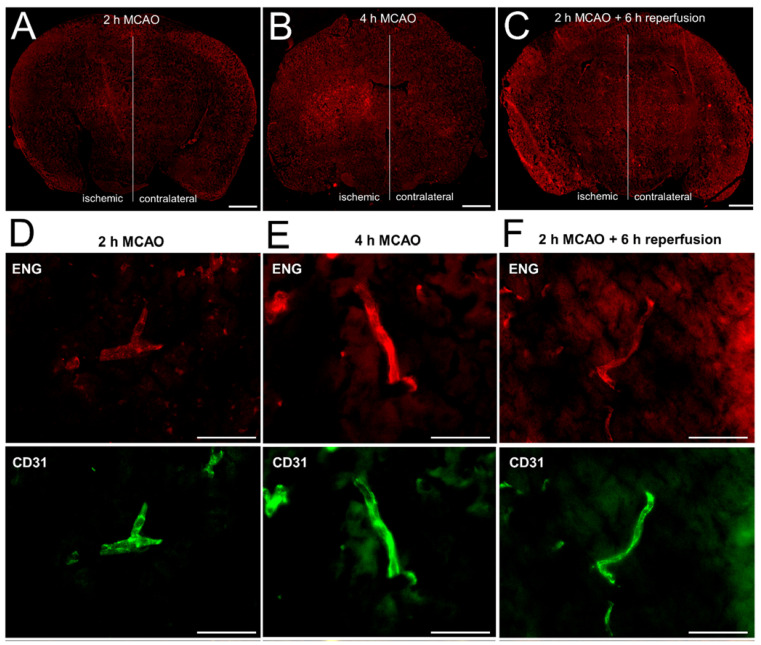

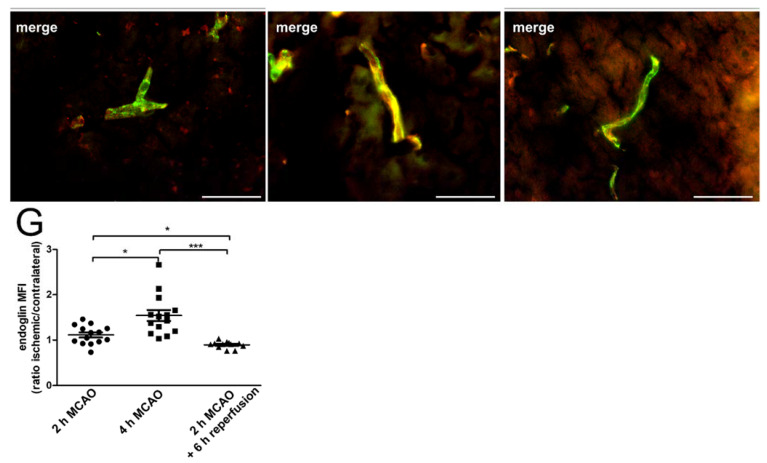
The upregulation and release of ENG in permanent versus transient middle cerebral artery occlusion (MCAO) in a murine stroke model. Representative immunohistochemistry of cerebral ENG after 2 h (**A**) and 4 h (**B**) of permanent MCAO or after 2 h of MCAO with an additional 6 h of reperfusion (**C**). Sections show tissue 0.5 mm anterior from bregma. Scale bar = 1000 µm. (**D**–**F**) Endothelial expression of ENG (red) on an individual vessel as identified by CD31 staining (green) of the basal ganglia region of the ischemic hemisphere in more detail. Scale bar = 50 µm. (**G**) Quantification of ENG expression in the basal ganglia depicted as the ratio of the mean fluorescence intensity between three corresponding regions of interest in ischemic vs. contralateral hemispheres, using ImageJ in *n* = 4 (2 h MCAO + 6 h reperfusion) and *n* = 5 (2 + 4 h MCAO) animals. (* *p* < 0.05; *** *p* < 0.001).

To corroborate our in vitro finding that the sequence of hypoxia followed by reoxygenation, but not hypoxia alone, induced shedding of ENG, we next investigated ENG expression in a murine stroke model. When examining MCAO in mice, immunohistochemistry revealed a time-dependent upregulation of ENG in the infarcted tissue in vivo (Figure 3A,B). In a third group, the MCAO was transient. Here, the occluding filament was removed after 2 h, allowing a reperfusion period of another 6 h that mimicked the situation after a mechanical thrombectomy. In accordance with ENG shedding after reoxygenation in vitro, ENG expression was significantly lower after transient MCAO (Figure 3C,G). This regulation can also be appreciated when looking at individual vessels in the infarct core, where ENG co-localizes with the endothelial marker CD31 (Figure 3D–F).

### 2.3. Soluble ENG Induces an Inflammatory Phenotype in Brain Endothelial Cells

**Figure 4:**
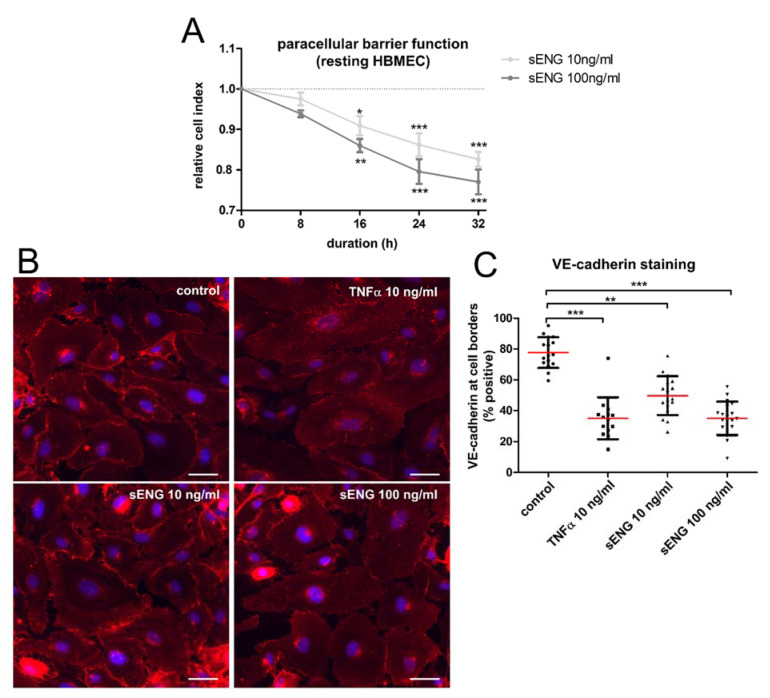
Soluble ENG impairs resting endothelial monolayer integrity causing increased paracellular permeability and the downregulation of VE-cadherin. (**A**) Label-free assessment of transendothelial resistance of HBMEC in an impedance-based xCELLigence DP system. Confluent monolayers were stimulated with the indicated concentrations of sENG. Data represent the changes relative to untreated cells of five independent experiments. (**B**) Immunocytochemistry of resting HBMEC visualizing VE-cadherin after stimulation, as indicated. Representative of three experiments. Scale bar = 50 µm. (**C**) Quantification of VE-cadherin staining by blotting the ratio of the cell borders with positive staining to the cell circumference. Statistical testing for normal distribution by the D’Agostino–Pearson omnibus K2 test, followed by one-way ANOVA and the Bonferroni post-hoc test. (* *p* < 0.05; ** *p* < 0.01; *** *p* < 0.001).

High levels of soluble ENG are reported in pre-eclampsia, which may be associated with cerebral edema and hemorrhage, which are, in turn, also seen in cerebral I/R-injury. Considering the significant shedding of ENG after hypoxia/reoxygenation, we looked for a possible autocrine stimulation and tested the effect of recombinant human sENG on the human brain endothelium. For the label-free real-time measurement of transendothelial resistance, we used an xCELLigence DP system. HBMEC were cultivated on gold-electrode-coated plates until they formed a confluent monolayer. Subsequently, the resting cells were stimulated with 10 or 100 ng/mL sENG. Both concentrations caused a significant and sustained reduction in endothelial barrier function, as shown by a decrease in the cell index (Figure 4A). Accordingly, immunocytochemistry of the resting brain endothelium showed an inflammatory phenotype after stimulation with sENG. There was a redistribution of VE-cadherin away from the cell borders and an increase in denuded areas, similar to TNFα stimulation (Figure 4B,C). In summary, sENG stimulation caused an inflammatory phenotype in the human brain endothelium and compromised barrier function.

## 3. Discussion

In the present study, we unravel the role of brain endothelial ENG by characterizing its cellular expression, as well as the release of sENG during hypoxia and subsequent reoxygenation, as a model of I/R-injury in cerebral stroke. As our principal finding, we show that ENG upregulation upon hypoxia can pass over into extensive shedding of its soluble isoform when followed by reoxygenation. Soluble ENG, in turn, causes an inflammatory phenotype in the human brain endothelium.

The presented ENG induction during hypoxia and in response to inflammatory stimuli is in line with data from other vascular beds [7,14,24]. Analogously, ENG is upregulated in hypoxic tissue in post-mortem sections of patients with stroke and in the ischemic brain tissue of mice [19,25]. In the acute phase, ENG upregulation potentially protects endothelia from TGFβ-induced apoptosis, and in the further course, its expression is a prerequisite for angiogenesis [7,26]. Thus, in the context of prolonged LVO, ENG upregulation may play a critical role in limiting tissue damage. Correspondingly, heterozygous ENG-deficient mice subjected to permanent MCAO had larger stroke volumes and were more severely impaired than wild-type animals [25].

With mechanical thrombectomy as a treatment option for acute LVO stroke, the restoration of cerebral blood flow fundamentally alters the locally prevailing milieu: as a consequence of recanalization, ENG that has been upregulated under occlusion is then extensively shed, resulting in the loss of membranous ENG and the generation of sENG. This may have several implications: as demonstrated, sENG directly modulates brain endothelial barrier function, causing an inflamed phenotype. This is in accordance with data in HUVEC showing that sENG is capable of inducing NF-κB translocation and the upregulation of vascular cell adhesion molecule-1 (VCAM-1). Additionally, mice injected with adenovirus containing sENG showed increased vascular permeability, as assessed by Evans blue leakage in the lungs and liver [27,28]. The ENG/sENG axis is regulated in various cerebrovascular diseases [29,30,31]. With respect to brain endothelial dysfunction, the most interesting data come from women with pre-eclampsia, an acute condition associated with cerebral edema and hemorrhage. Here, sENG concentrations of about 35–100 ng/mL, in the range of our in vitro effects, have been shown to correlate with disease severity [27].

More recently, ENG has been shown to also serve as a ligand to integrin αIIbβ3 on platelets via the binding of its RGD motif, mediating endothelial adhesion and causing the activation of platelets, as represented by filopodia and lamellipodia formation [5]. Furthermore, ENG can interact with integrin α5β1 on leukocytes, particularly in the presence of the inflammatory chemokine CXCL12 [32]. Thus, besides a direct effect on endothelial barrier function, the regulation of endothelial ENG expression and sENG release may also have further implications for the modulation of detrimental intravascular platelet–leukocyte interactions that have previously been identified as key orchestrators of cerebral I/R-injury [33,34]. In principle, our discovery of a vasoactive substance released after hypoxia/reoxygenation may explain both the delayed and the progressive endothelial dysfunction that occur in some stroke patients after a mechanical thrombectomy. The recent detection of rt-PA in pial blood samples after LVO, showing collateral flow in the penumbra, suggests that there is the potential for complementary acute stroke therapies, including the inhibition of sENG shedding and the application of a scavenging antibody prior to recanalization [35], to mitigate I/R-injury and address a currently unmet need in stroke therapy. Thus, to further prove the pathophysiological relevance of our findings, the evaluation of sENG as a biomarker or therapeutic target requires further investigation in stroke patients subjected to mechanical thrombectomy.

Collectively, our data indicate that sENG is extensively generated upon hypoxia/reoxygenation and functions as a direct inflammatory mediator at the brain endothelium, pointing towards a potential contribution of the ENG/sENG axis to reperfusion injury in cerebral stroke.

## 4. Materials and Methods

### 4.1. Cell Culture

Human brain endothelial cells (HBMEC) were purchased from Cell Systems (ACBRI 376, Kirkland, WA 98034, USA) and cultured at 37 °C/5% CO_2_ in gelatin-coated flasks in RPMI-1640 medium (Sigma-Aldrich, St. Louis, MO, USA) supplemented exactly as formerly described [36]. The cells did not exceed passage 9. Recombinant human ENG was purchased from R&D Systems (1097-EN, Minneapolis, MN, USA) and solved in PBS as recommended by the manufacturer.

### 4.2. Western Blotting

Whole-cell protein extracts were prepared as formerly described [37]. Primary antibodies against ENG (1:1000, #14606, Cell Signal, Danvers, MA, USA) and GAPDH (1:1000, Santa Cruz Biotechnology, Dallas, TX, USA) were combined with appropriate peroxidase-coupled secondary antibodies (Jackson ImmunoResearch, West Grove, PA, USA) and employed with an enhanced chemoluminescence system (Perkin Elmer, Waltham, MA, USA).

### 4.3. Quantitative Real-Time PCR

For RNA isolation, cells in culture flasks were rinsed with ice-cold PBS, scrapped in 1 mL of Trizol reagent (Invitrogen, Waltham, MA, USA), and vigorously vortexed. After a 5 min incubation at room temperature, the samples were incubated with 200 µL of chloroform and mixed by vigorous shaking. After another 3 min of incubation at room temperature, the tubes were centrifuged (14,000 rpm) at 4 °C for 15 min. Subsequently, 500 µL of the upper clean phase was transferred to a new tube and mixed with 500 µL isopropanol. After 10 min of incubation at room temperature and another centrifugation step, the supernatant was removed, and the remaining pellet was washed with ice-cold 75% ethanol. After washing and centrifugation, the supernatant was discarded completely, and the pellet was air-dried and re-suspended in 30 µL DEPC-H_2_O. To ensure even RNA quantities, we measured the RNA concentrations by a photometer prior to cDNA synthesis using the TaqMan^®^ Reverse Transcription kit (Applied Biosystems, Carlsbad, CA, USA) and subjected them in triplicates to quantitative real-time PCR using TaqMan^®^ reagents and the FAM-MBG inventoried primers, namely Hs00923996_m1 (ENG, mean CT-value: 237), Hs01037003_g1 (MMP14, mean CT-value: 296), and 02786624_g1 (GAPDH, mean CT-value: 138), in a StepOne plus system (all from Applied Biosystems, Carlsbad, CA, USA). A total of 40 cycles were run with a cycling stage of 95 °C for 15 s followed by 60 °C for 60 s. Relative changes in gene expression were normalized to the housekeeping gene as an internal control.

### 4.4. xCELLigence Assay

Label-free real-time assessment of transendothelial resistance was performed in an xCELLigence DP system (San Diego, CA, USA), which records the impedance changes compared to the background of cell-free electrodes at three different alternating current frequencies expressed as the dimensionless Cell Index (CI). The Cell Index is defined as: CI = (impedance at time point n − impedance in the absence of cells)/nominal impedance value [38]. The CI correlates to the transendothelial electrical resistance but additionally reflects the capacitance of the cell layer. When confluent, as indicated by a plateau of the CI, resting cells were stimulated in duplicates with recombinant human sENG.

### 4.5. Immunocytochemistry

Cells were cultured on 24-well plates coated with rat collagen (100 µg/mL) until they formed confluent monolayers. After stimulation, cells were rinsed and fixed/permeabilized with 3.7% paraformaldehyde and 0.1% Triton X-100 for 10 min. After the washing steps, a monoclonal (F-8) mouse anti-VE-cadherin antibody (1:250, SC-9989, Santa Cruz Biotechnologies, Dallas, TX, USA) was added for 1 h at room temperature. Subsequently, a corresponding secondary antibody was incubated for another 1 h. An anti-fading agent was added. Images were taken with a Leica DMi8 inverted microscope (Wetzlar, Germany). VE-cadherin staining was quantified by dividing the cell circumference by the length of VE-cadherin staining at the cell border (% positive) using ImageJ(version 1.53k by Wayne Rasband and contributors, National Institutes of Health, Bethesda, MD, USA; https://imagej.nih.gov/ij/download.html, accessed on 27 May 2022).

### 4.6. Animals

Animal studies were approved by the district government of lower Franconia and were conducted in accordance with the U.S. National Institutes of Health Guide for the Care and Use of Laboratory Animals. The experiments were designed, performed, and reported according to the Animal Research: Reporting of In Vivo Experiments guidelines [39]. All C57Bl/6 N mice (6–8 weeks old) were purchased from Charles River Laboratories (Sulzfeld, Germany). We randomized the mice and subjected them to a permanent or transient middle cerebral artery occlusion (MCAO).

### 4.7. Ischemia Model

Focal cerebral ischemia was induced by a 2 h and 4 h MCAO or a 2 h MCAO with a 6 h reperfusion phase [40]. Mice for all animal experiments were randomized and coded by an independent researcher who was not involved in data analysis, so that experiments were carried out blindly. Investigators involved in the surgery and evaluation of all readout parameters were blinded to the experimental groups. To reduce the variability of our outcome parameters caused by sex differences and to decrease group sizes, we used only male mice in the study. Mice were excluded from endpoint analyses for the following pre-specified reasons: (1) death before the predefined experimental endpoint (in this study *n* = 1/15; 6,7%); (2) drop-out score (weight loss, general condition, spontaneous behavior); (3) operation time > 10 min (to exclude the influence of prolonged anesthesia and increase group comparability). For the induction of MCAO, mice were anesthetized with 2% isoflurane in O_2_ (*v*/*v*), and we injected 200 mg/kg of body weight Metamizol subcutaneously. Lidocaine gel was used on the margin of the wound as analgesia. To maintain a core body temperature close to 37 °C throughout surgery, a servo-controlled heating blanket was used. After a midline neck incision, a standardized silicon rubber-coated no. 6.0 nylon monofilament (6023910PK10, Doccol, Sharon, MA, USA) was inserted into the right common carotid artery and advanced via the internal carotid artery to occlude the origin of the MCA for the different durations. Animals were either sacrificed after 2 h or 4 h or re-anesthetized to remove the occluding filament after 2 h, allowing reperfusion for another 6 h. For a timeline, see Supplementary Figure S2.

### 4.8. Immunohistochemistry

For immunohistochemistry, mice were anesthetized with isoflurane and sacrificed by decapitation. Brain tissue was cut into 2 mm-thick coronal sections, embedded in Tissue-Tek OCT compound, and frozen. For ENG staining, the cryo-embedded brains were cut into 10 μm-thick slices and fixed in 4% PFA in PBS; the blocking of epitopes was achieved by pre-treatment with 5% bovine serum albumin (BSA) and 0.2% Triton X-100 in PBS for 60 min to prevent unspecific binding. For the staining of ENG, rabbit recombinant anti-CD105 antibody (ab221675, abcam, Cambridge, UK) at a dilution of 1:50 in PBS containing 1% BSA was added overnight at 4 °C. Afterwards, slides were incubated with a goat anti-rabbit IgG secondary antibody coupled with Alexa Fluor 594 (A-11012, Thermo Fisher Scientific, Waltham, MA, USA) at a dilution of 1:100 in PBS containing 1% BSA for 1 h. For the quantification of ENG, MFI identical brain sections at the level of the basal ganglia (0.5 mm anterior from bregma) were selected, and imaging was performed for 4–5 different animals in each group under a microscope (Leica DMi8 equipped with the DFC 3000 G camera control and LAS X software (Leica, Wetzlar, Germany)).

### 4.9. Statistics

Statistical analysis was performed using GraphPad PRISM Version 5.04 (La Jolla, CA, USA). For the comparison of multiple groups, we performed the non-parametric Kruskal–Wallis test followed by Dunn’s post-hoc test. Alternatively, when normal distribution testing by the D’Agostino–Pearson omnibus K2 test was passed, we performed a one-way ANOVA and the Bonferroni post-hoc test. *p* values < 0.05 were considered statistically significant. Asterisks indicate levels of significance (* *p* < 0.05; ** *p* < 0.01; *** *p* < 0.001).

## Data Availability

The datasets used and/or analyzed during the current study are available from the corresponding author on reasonable request.

## Supplementary Materials

The following supporting information can be downloaded at: https://www.mdpi.com/article/10.3390/ijms23137085/s1.

## Abbreviations

large vessel occlusion (LVO), middle cerebral artery occlusion (MCAO), ischemia/reperfusion (I/R), endoglin (ENG), soluble endoglin (sENG) human umbilical cord vein endothelial cells (HUVEC), human brain microvascular endothelial cells (HBMEC), mechanical thrombectomy (MT), blood-brain barrier (BBB), cell index (CI), matrix metalloproteinase-12 and -14 (MMP12 and MMP14, respectively).
