# Supplementary material for: Regulation and Release of Vasoactive Endoglin by Brain Endothelium in Response to Hypoxia/Reoxygenation in Stroke
Source: Int J Mol Sci. 2022 Jun 25;23(13):7085. doi: 10.3390/ijms23137085 (PMC9267030; doi:10.3390/ijms23137085)
Supplement: Supplementary file 1 [file ijms-23-07085-s001.zip › ijms-1769370-supplementary.pdf]

Supplementary Materials:

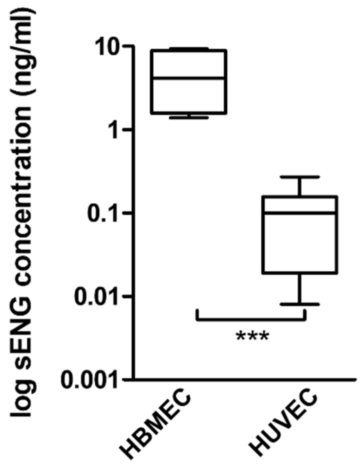

Figure S1. Comparative soluble endoglin concentrations in supernatants of human brain microvascular endothelial cells (left) and human umbilical vein endothelial cells (right). Note that the y-axis is on log scale.

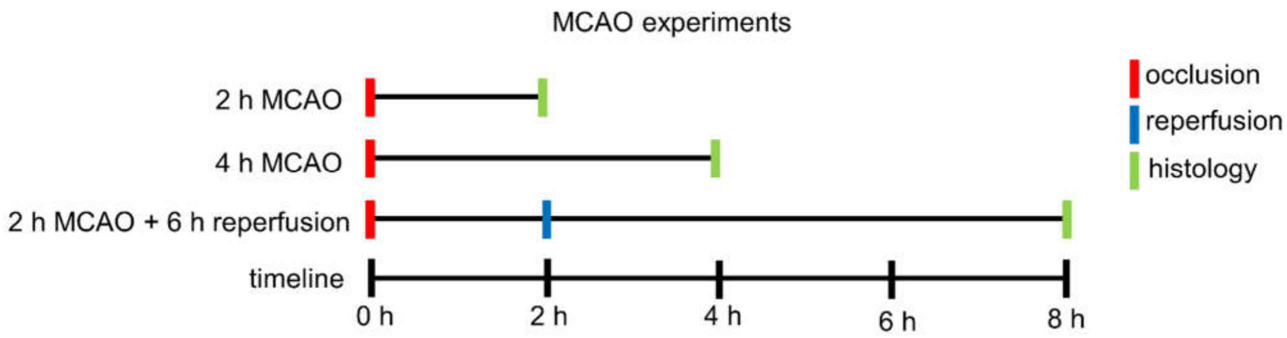

Figure S2. Timeline of the animal experiments.
